# Di-*n*-butyl­bis­(thio­cyanato-κ*N*)(1,10-phenanthroline-κ^2^ *N*,*N*′)tin(IV)

**DOI:** 10.1107/S1600536811001292

**Published:** 2011-01-15

**Authors:** Ezzatollah Najafi, Mostafa M. Amini, Seik Weng Ng

**Affiliations:** a Department of Chemistry, General Campus, Shahid Beheshti University, Tehran 1983963113, Iran; b Department of Chemistry, University of Malaya, 50603 Kuala Lumpur, Malaysia

## Abstract

In the asymmetric unit of the title compound, [Sn(C_4_H_9_)_2_(NCS)_2_(C_12_H_8_N_2_)], there are two independent mol­ecules, both lying on a twofold rotation axis. The axis passes through the mid-point of the 1,10 and 5,6 bonds of the *N*-heterocycle and through the Sn atom. The Sn atoms show a slightly distorted SnC_2_N_4_ octa­hedral coordination.

## Related literature

For the di-*n*-butyl­tin dichloride adduct, see: Ganis *et al.* (1983 ▶).
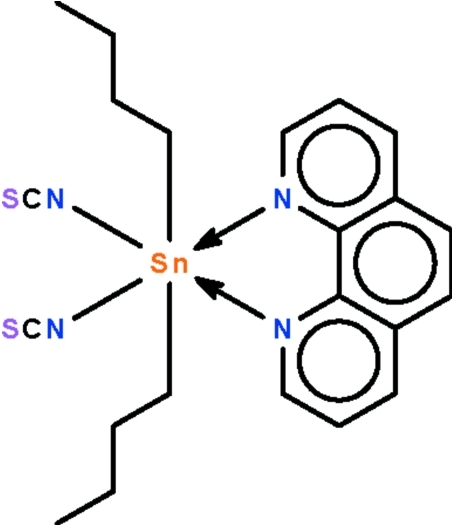


## Experimental

### 

#### Crystal data

[Sn(C_4_H_9_)_2_(NCS)_2_(C_12_H_8_N_2_]
*M* *_r_* = 529.28
Monoclinic, 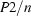

*a* = 15.0008 (3) Å
*b* = 10.5220 (2) Å
*c* = 15.8359 (3) Å
β = 107.452 (2)°
*V* = 2384.46 (8) Å^3^
*Z* = 4
Mo *K*α radiation
μ = 1.26 mm^−1^
*T* = 100 K
0.30 × 0.20 × 0.10 mm

#### Data collection

Agilent Technologies SuperNova diffractometer with an Atlas detector
Absorption correction: multi-scan (*CrysAlis PRO*; Agilent Technologies, 2010 ▶) *T*_min_ = 0.703, *T*_max_ = 0.884
11981 measured reflections
5323 independent reflections
4659 reflections with *I* > 2σ(*I*)
*R*_int_ = 0.029

#### Refinement

*R*[*F* ^2^ > 2σ(*F* ^2^)] = 0.029
*wR*(*F* ^2^) = 0.070
*S* = 1.02
5323 reflections
263 parameters
H-atom parameters constrained
Δρ_max_ = 0.43 e Å^−3^
Δρ_min_ = −0.61 e Å^−3^

### 

Data collection: *CrysAlis PRO* (Agilent Technologies, 2010 ▶); cell refinement: *CrysAlis PRO*; data reduction: *CrysAlis PRO*; program(s) used to solve structure: *SHELXS97* (Sheldrick, 2008 ▶); program(s) used to refine structure: *SHELXL97* (Sheldrick, 2008 ▶); molecular graphics: *X-SEED* (Barbour, 2001 ▶); software used to prepare material for publication: *publCIF* (Westrip, 2010 ▶).

## Supplementary Material

Crystal structure: contains datablocks global, I. DOI: 10.1107/S1600536811001292/bt5460sup1.cif

Structure factors: contains datablocks I. DOI: 10.1107/S1600536811001292/bt5460Isup2.hkl

Additional supplementary materials:  crystallographic information; 3D view; checkCIF report

## Figures and Tables

**Fig. 1.**
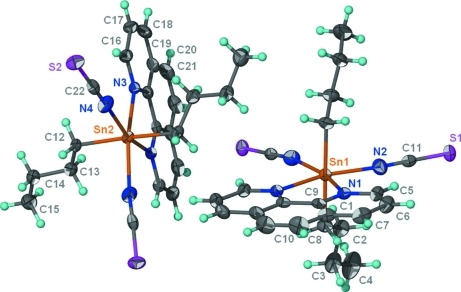
Anisotropic displacement ellipsoid plot (Barbour, 2001) of the two independent molecules of dibutyldiisothiocyanato(1,10-phenanthroline)tin at the 70% probability level; hydrogen atoms are drawn as spheres of arbitrary radius.

## supplementary crystallographic information

### Comment

Diorganotin dihalides/pseudohalides form a number of adducts with 1,10-phenanthroline. The dihalides adducts have been better studied, particularly with dibutyltin dihalides adducts; the di-*n*-butyltin dichloride adduct was reported a long time ago (Ganis *et al.*, 1983). The diisothiocyanate adduct (Scheme I, Fig. 1 & 2), also features the chelated tin atom in an octahedral geometry. The two independent molecules both lie on a twofold rotation axis; the axis passes through the mid-point of the 1,10 and 5,6 pairs of atoms of the *N*-heterocycle, and it relates one butyl group to the other (as well as one isothiocyanate group to the other).

### Experimental

Dibutyltin diisothiocyanate and 1,10-phenanthroline (1 mmol) were loaded into a convection tube. The tube was filled with dry methanol and kept at 333 K. Colorless crystals were collected from the side arm after several days.

### Refinement

H-atoms were placed in calculated positions [C—H 0.95 to 0.98 Å, *U*_iso_(H) 1.2 to 1.5*U*_eq_(C)] and were included in the refinement in the riding model approximation.

### Crystal data

**Table:**

[Sn(C_4_H_9_)_2_(NCS)_2_(C_12_H_8_N_2_]	*F*(000) = 1072
*M**_r_* = 529.28	*D*_x_ = 1.474 Mg m^−^^3^
Monoclinic, *P*2/*n*	Mo *K*α radiation, λ = 0.71073 Å
Hall symbol: -P 2yac	Cell parameters from 10240 reflections
*a* = 15.0008 (3) Å	θ = 2.2–29.4°
*b* = 10.5220 (2) Å	µ = 1.26 mm^−^^1^
*c* = 15.8359 (3) Å	*T* = 100 K
β = 107.452 (2)°	Prism, colorless
*V* = 2384.46 (8) Å^3^	0.30 × 0.20 × 0.10 mm
*Z* = 4	

### Data collection

**Table:**

Agilent Technologies SuperNova (Dual, Cu at zero) diffractometer with an Atlas detector	5323 independent reflections
Radiation source: SuperNova (Mo) X-ray Source	4659 reflections with *I* > 2σ(*I*)
Mirror	*R*_int_ = 0.029
Detector resolution: 10.4041 pixels mm^-1^	θ_max_ = 27.5°, θ_min_ = 2.2°
ω scans	*h* = −19→18
Absorption correction: multi-scan *CrysAlis PRO* (Agilent Technologies, 2010)	*k* = −13→13
*T*_min_ = 0.703, *T*_max_ = 0.884	*l* = −13→20
11981 measured reflections	

### Refinement

**Table:**

Refinement on *F*^2^	Primary atom site location: structure-invariant direct methods
Least-squares matrix: full	Secondary atom site location: difference Fourier map
*R*[*F*^2^ > 2σ(*F*^2^)] = 0.029	Hydrogen site location: inferred from neighbouring sites
*wR*(*F*^2^) = 0.070	H-atom parameters constrained
*S* = 1.02	*w* = 1/[σ^2^(*F*_o_^2^) + (0.0326*P*)^2^ + 0.2915*P*] where *P* = (*F*_o_^2^ + 2*F*_c_^2^)/3
5323 reflections	(Δ/σ)_max_ = 0.001
263 parameters	Δρ_max_ = 0.43 e Å^−^^3^
0 restraints	Δρ_min_ = −0.61 e Å^−^^3^

### Fractional atomic coordinates and isotropic or equivalent isotropic displacement parameters (Å^2^)

**Table:**

	*x*	*y*	*z*	*U*_iso_*/*U*_eq_	
Sn1	0.7500	0.646740 (19)	0.2500	0.01691 (7)	
Sn2	0.7500	0.61402 (2)	0.7500	0.01760 (7)	
S1	0.80427 (5)	0.90208 (6)	0.00369 (4)	0.02442 (15)	
S2	1.01982 (5)	0.34705 (6)	0.92845 (5)	0.02964 (16)	
N1	0.76659 (13)	0.46325 (17)	0.16982 (12)	0.0186 (4)	
N2	0.76347 (15)	0.7767 (2)	0.14184 (14)	0.0296 (5)	
N3	0.83931 (12)	0.79303 (17)	0.80419 (12)	0.0168 (4)	
N4	0.87303 (16)	0.4872 (2)	0.82567 (14)	0.0328 (5)	
C1	0.60205 (16)	0.6504 (2)	0.19698 (17)	0.0232 (5)	
H1A	0.5832	0.5919	0.1457	0.028*	
H1B	0.5745	0.6180	0.2423	0.028*	
C2	0.56148 (17)	0.7817 (2)	0.16729 (18)	0.0291 (6)	
H2A	0.5763	0.8055	0.1125	0.035*	
H2B	0.5917	0.8446	0.2135	0.035*	
C3	0.45576 (19)	0.7876 (3)	0.14991 (18)	0.0394 (7)	
H3A	0.4255	0.7214	0.1063	0.047*	
H3B	0.4409	0.7696	0.2056	0.047*	
C4	0.4165 (2)	0.9175 (3)	0.1149 (2)	0.0578 (10)	
H4A	0.3488	0.9181	0.1049	0.087*	
H4B	0.4297	0.9346	0.0590	0.087*	
H4C	0.4458	0.9831	0.1582	0.087*	
C5	0.78497 (16)	0.4644 (2)	0.09300 (15)	0.0238 (5)	
H5	0.7912	0.5440	0.0670	0.029*	
C6	0.79562 (19)	0.3532 (2)	0.04866 (17)	0.0292 (6)	
H6	0.8094	0.3574	−0.0060	0.035*	
C7	0.78590 (17)	0.2385 (2)	0.08520 (17)	0.0303 (6)	
H7	0.7913	0.1621	0.0551	0.036*	
C8	0.76801 (16)	0.2333 (2)	0.16709 (17)	0.0246 (6)	
C9	0.75910 (15)	0.3498 (2)	0.20787 (15)	0.0184 (5)	
C10	0.7587 (2)	0.1170 (2)	0.21069 (19)	0.0354 (7)	
H10	0.7649	0.0383	0.1836	0.042*	
C11	0.78116 (16)	0.8286 (2)	0.08429 (15)	0.0191 (5)	
C12	0.70141 (16)	0.5978 (2)	0.86263 (15)	0.0207 (5)	
H12A	0.6957	0.5064	0.8749	0.025*	
H12B	0.7494	0.6343	0.9142	0.025*	
C13	0.60877 (17)	0.6616 (2)	0.85540 (16)	0.0245 (5)	
H13A	0.5619	0.6324	0.8004	0.029*	
H13B	0.6162	0.7545	0.8505	0.029*	
C14	0.57178 (19)	0.6351 (2)	0.93319 (17)	0.0289 (6)	
H14A	0.5663	0.5421	0.9397	0.035*	
H14B	0.6171	0.6675	0.9881	0.035*	
C15	0.47828 (19)	0.6955 (3)	0.92244 (19)	0.0389 (7)	
H15A	0.4576	0.6755	0.9740	0.058*	
H15B	0.4327	0.6626	0.8688	0.058*	
H15C	0.4836	0.7879	0.9175	0.058*	
C16	0.92697 (16)	0.7912 (2)	0.85792 (15)	0.0217 (5)	
H16	0.9561	0.7116	0.8764	0.026*	
C17	0.97730 (18)	0.9027 (2)	0.88790 (16)	0.0262 (6)	
H17	1.0397	0.8983	0.9260	0.031*	
C18	0.93621 (17)	1.0177 (2)	0.86206 (16)	0.0262 (6)	
H18	0.9701	1.0939	0.8815	0.031*	
C19	0.84303 (17)	1.0231 (2)	0.80631 (16)	0.0224 (5)	
C20	0.79742 (15)	0.9072 (2)	0.77864 (14)	0.0170 (5)	
C21	0.79440 (18)	1.1396 (2)	0.77720 (18)	0.0282 (6)	
H21	0.8249	1.2182	0.7964	0.034*	
C22	0.93256 (18)	0.4328 (2)	0.86806 (16)	0.0214 (5)	

### Atomic displacement parameters (Å^2^)

**Table:**

	*U*^11^	*U*^22^	*U*^33^	*U*^12^	*U*^13^	*U*^23^
Sn1	0.01948 (13)	0.01283 (11)	0.01867 (12)	0.000	0.00609 (9)	0.000
Sn2	0.01997 (13)	0.01451 (12)	0.01626 (12)	0.000	0.00229 (9)	0.000
S1	0.0332 (4)	0.0204 (3)	0.0224 (3)	0.0006 (3)	0.0125 (3)	0.0011 (2)
S2	0.0255 (3)	0.0243 (3)	0.0335 (4)	0.0060 (3)	0.0005 (3)	0.0004 (3)
N1	0.0181 (10)	0.0161 (9)	0.0203 (10)	0.0001 (8)	0.0039 (8)	−0.0006 (8)
N2	0.0296 (12)	0.0284 (11)	0.0316 (12)	0.0009 (10)	0.0105 (10)	0.0061 (10)
N3	0.0178 (9)	0.0175 (9)	0.0155 (9)	0.0000 (8)	0.0056 (8)	−0.0003 (8)
N4	0.0346 (13)	0.0373 (13)	0.0237 (11)	0.0053 (11)	0.0043 (10)	0.0009 (10)
C1	0.0183 (12)	0.0224 (12)	0.0282 (13)	0.0032 (10)	0.0058 (10)	−0.0001 (10)
C2	0.0286 (14)	0.0261 (13)	0.0307 (14)	0.0108 (11)	0.0062 (12)	0.0040 (11)
C3	0.0334 (15)	0.0569 (19)	0.0293 (15)	0.0216 (14)	0.0117 (12)	0.0055 (14)
C4	0.059 (2)	0.075 (2)	0.0418 (19)	0.044 (2)	0.0178 (17)	0.0180 (18)
C5	0.0262 (13)	0.0254 (12)	0.0188 (12)	0.0032 (11)	0.0052 (10)	0.0015 (10)
C6	0.0326 (14)	0.0309 (14)	0.0253 (14)	0.0074 (12)	0.0104 (12)	−0.0034 (11)
C7	0.0290 (14)	0.0266 (13)	0.0341 (15)	0.0083 (11)	0.0076 (12)	−0.0107 (12)
C8	0.0229 (12)	0.0172 (11)	0.0320 (14)	0.0025 (10)	0.0055 (11)	−0.0055 (10)
C9	0.0123 (11)	0.0167 (11)	0.0246 (13)	−0.0002 (9)	0.0030 (10)	−0.0002 (9)
C10	0.0415 (17)	0.0161 (11)	0.0488 (18)	0.0020 (12)	0.0139 (15)	−0.0046 (12)
C11	0.0187 (12)	0.0148 (10)	0.0220 (12)	0.0013 (9)	0.0035 (10)	−0.0045 (10)
C12	0.0234 (12)	0.0228 (12)	0.0154 (11)	−0.0037 (10)	0.0050 (10)	0.0027 (10)
C13	0.0246 (13)	0.0272 (13)	0.0211 (12)	−0.0022 (11)	0.0060 (11)	0.0017 (10)
C14	0.0300 (14)	0.0351 (14)	0.0216 (13)	−0.0056 (12)	0.0078 (11)	−0.0002 (11)
C15	0.0296 (15)	0.0591 (19)	0.0305 (15)	−0.0067 (14)	0.0128 (12)	−0.0019 (14)
C16	0.0209 (12)	0.0244 (12)	0.0193 (12)	0.0000 (10)	0.0054 (10)	−0.0028 (10)
C17	0.0213 (12)	0.0344 (14)	0.0218 (13)	−0.0055 (11)	0.0050 (10)	−0.0078 (11)
C18	0.0286 (13)	0.0237 (12)	0.0307 (14)	−0.0119 (11)	0.0154 (11)	−0.0097 (11)
C19	0.0265 (13)	0.0209 (11)	0.0248 (12)	−0.0033 (10)	0.0151 (11)	−0.0025 (10)
C20	0.0206 (12)	0.0162 (11)	0.0167 (11)	−0.0008 (9)	0.0092 (10)	−0.0022 (9)
C21	0.0358 (15)	0.0170 (11)	0.0386 (16)	−0.0042 (10)	0.0217 (13)	−0.0037 (10)
C22	0.0305 (14)	0.0147 (11)	0.0240 (13)	−0.0035 (11)	0.0157 (11)	−0.0056 (10)

### Geometric parameters (Å, °)

**Table:**

Sn1—C1^i^	2.125 (2)	C5—H5	0.9500
Sn1—C1	2.125 (2)	C6—C7	1.365 (4)
Sn1—N2^i^	2.248 (2)	C6—H6	0.9500
Sn1—N2	2.248 (2)	C7—C8	1.402 (4)
Sn1—N1	2.3646 (19)	C7—H7	0.9500
Sn1—N1^i^	2.3646 (19)	C8—C9	1.410 (3)
Sn2—C12^ii^	2.126 (2)	C8—C10	1.433 (4)
Sn2—C12	2.126 (2)	C9—C9^i^	1.440 (5)
Sn2—N4^ii^	2.302 (2)	C10—C10^i^	1.347 (6)
Sn2—N4	2.302 (2)	C10—H10	0.9500
Sn2—N3	2.3215 (18)	C12—C13	1.516 (3)
Sn2—N3^ii^	2.3215 (18)	C12—H12A	0.9900
S1—C11	1.616 (3)	C12—H12B	0.9900
S2—C22	1.641 (3)	C13—C14	1.521 (4)
N1—C5	1.326 (3)	C13—H13A	0.9900
N1—C9	1.357 (3)	C13—H13B	0.9900
N2—C11	1.159 (3)	C14—C15	1.502 (4)
N3—C16	1.335 (3)	C14—H14A	0.9900
N3—C20	1.360 (3)	C14—H14B	0.9900
N4—C22	1.102 (3)	C15—H15A	0.9800
C1—C2	1.526 (3)	C15—H15B	0.9800
C1—H1A	0.9900	C15—H15C	0.9800
C1—H1B	0.9900	C16—C17	1.399 (3)
C2—C3	1.527 (4)	C16—H16	0.9500
C2—H2A	0.9900	C17—C18	1.364 (3)
C2—H2B	0.9900	C17—H17	0.9500
C3—C4	1.525 (4)	C18—C19	1.413 (3)
C3—H3A	0.9900	C18—H18	0.9500
C3—H3B	0.9900	C19—C20	1.403 (3)
C4—H4A	0.9800	C19—C21	1.430 (3)
C4—H4B	0.9800	C20—C20^ii^	1.440 (4)
C4—H4C	0.9800	C21—C21^ii^	1.352 (5)
C5—C6	1.398 (3)	C21—H21	0.9500
			
C1^i^—Sn1—C1	177.93 (12)	N1—C5—C6	122.7 (2)
C1^i^—Sn1—N2^i^	90.57 (9)	N1—C5—H5	118.7
C1—Sn1—N2^i^	88.18 (8)	C6—C5—H5	118.7
C1^i^—Sn1—N2	88.18 (8)	C7—C6—C5	119.0 (3)
C1—Sn1—N2	90.57 (9)	C7—C6—H6	120.5
N2^i^—Sn1—N2	105.05 (11)	C5—C6—H6	120.5
C1^i^—Sn1—N1	87.78 (8)	C6—C7—C8	120.1 (2)
C1—Sn1—N1	93.91 (8)	C6—C7—H7	120.0
N2^i^—Sn1—N1	162.54 (7)	C8—C7—H7	120.0
N2—Sn1—N1	92.27 (7)	C7—C8—C9	117.4 (2)
C1^i^—Sn1—N1^i^	93.91 (8)	C7—C8—C10	123.5 (2)
C1—Sn1—N1^i^	87.78 (8)	C9—C8—C10	119.1 (2)
N2^i^—Sn1—N1^i^	92.27 (7)	N1—C9—C8	122.0 (2)
N2—Sn1—N1^i^	162.54 (7)	N1—C9—C9^i^	118.39 (13)
N1—Sn1—N1^i^	70.53 (9)	C8—C9—C9^i^	119.65 (15)
C12^ii^—Sn2—C12	170.80 (13)	C10^i^—C10—C8	121.30 (15)
C12^ii^—Sn2—N4^ii^	86.55 (8)	C10^i^—C10—H10	119.4
C12—Sn2—N4^ii^	88.12 (8)	C8—C10—H10	119.4
C12^ii^—Sn2—N4	88.12 (8)	N2—C11—S1	179.1 (2)
C12—Sn2—N4	86.55 (8)	C13—C12—Sn2	115.99 (15)
N4^ii^—Sn2—N4	109.11 (11)	C13—C12—H12A	108.3
C12^ii^—Sn2—N3	94.02 (8)	Sn2—C12—H12A	108.3
C12—Sn2—N3	93.44 (8)	C13—C12—H12B	108.3
N4^ii^—Sn2—N3	161.22 (7)	Sn2—C12—H12B	108.3
N4—Sn2—N3	89.68 (7)	H12A—C12—H12B	107.4
C12^ii^—Sn2—N3^ii^	93.44 (8)	C12—C13—C14	114.0 (2)
C12—Sn2—N3^ii^	94.02 (8)	C12—C13—H13A	108.8
N4^ii^—Sn2—N3^ii^	89.68 (7)	C14—C13—H13A	108.8
N4—Sn2—N3^ii^	161.22 (7)	C12—C13—H13B	108.8
N3—Sn2—N3^ii^	71.54 (9)	C14—C13—H13B	108.8
C5—N1—C9	118.9 (2)	H13A—C13—H13B	107.6
C5—N1—Sn1	124.72 (15)	C15—C14—C13	112.7 (2)
C9—N1—Sn1	116.34 (15)	C15—C14—H14A	109.1
C11—N2—Sn1	168.4 (2)	C13—C14—H14A	109.1
C16—N3—C20	118.75 (19)	C15—C14—H14B	109.1
C16—N3—Sn2	124.96 (15)	C13—C14—H14B	109.1
C20—N3—Sn2	116.29 (14)	H14A—C14—H14B	107.8
C22—N4—Sn2	173.9 (2)	C14—C15—H15A	109.5
C2—C1—Sn1	114.26 (17)	C14—C15—H15B	109.5
C2—C1—H1A	108.7	H15A—C15—H15B	109.5
Sn1—C1—H1A	108.7	C14—C15—H15C	109.5
C2—C1—H1B	108.7	H15A—C15—H15C	109.5
Sn1—C1—H1B	108.7	H15B—C15—H15C	109.5
H1A—C1—H1B	107.6	N3—C16—C17	122.2 (2)
C1—C2—C3	112.9 (2)	N3—C16—H16	118.9
C1—C2—H2A	109.0	C17—C16—H16	118.9
C3—C2—H2A	109.0	C18—C17—C16	119.5 (2)
C1—C2—H2B	109.0	C18—C17—H17	120.2
C3—C2—H2B	109.0	C16—C17—H17	120.2
H2A—C2—H2B	107.8	C17—C18—C19	119.8 (2)
C4—C3—C2	111.7 (3)	C17—C18—H18	120.1
C4—C3—H3A	109.3	C19—C18—H18	120.1
C2—C3—H3A	109.3	C20—C19—C18	117.3 (2)
C4—C3—H3B	109.3	C20—C19—C21	119.4 (2)
C2—C3—H3B	109.3	C18—C19—C21	123.3 (2)
H3A—C3—H3B	107.9	N3—C20—C19	122.4 (2)
C3—C4—H4A	109.5	N3—C20—C20^ii^	117.94 (12)
C3—C4—H4B	109.5	C19—C20—C20^ii^	119.61 (14)
H4A—C4—H4B	109.5	C21^ii^—C21—C19	121.02 (14)
C3—C4—H4C	109.5	C21^ii^—C21—H21	119.5
H4A—C4—H4C	109.5	C19—C21—H21	119.5
H4B—C4—H4C	109.5	N4—C22—S2	177.7 (2)
			
C1^i^—Sn1—N1—C5	−83.32 (19)	C6—C7—C8—C9	1.1 (4)
C1—Sn1—N1—C5	95.49 (18)	C6—C7—C8—C10	−178.7 (3)
N2^i^—Sn1—N1—C5	−168.1 (2)	C5—N1—C9—C8	−1.6 (3)
N2—Sn1—N1—C5	4.76 (18)	Sn1—N1—C9—C8	−179.73 (16)
N1^i^—Sn1—N1—C5	−178.3 (2)	C5—N1—C9—C9^i^	178.8 (2)
C1^i^—Sn1—N1—C9	94.70 (16)	Sn1—N1—C9—C9^i^	0.7 (3)
C1—Sn1—N1—C9	−86.49 (16)	C7—C8—C9—N1	0.5 (3)
N2^i^—Sn1—N1—C9	9.9 (3)	C10—C8—C9—N1	−179.6 (2)
N2—Sn1—N1—C9	−177.21 (15)	C7—C8—C9—C9^i^	−179.9 (3)
N1^i^—Sn1—N1—C9	−0.24 (11)	C10—C8—C9—C9^i^	0.0 (4)
C1^i^—Sn1—N2—C11	51.6 (10)	C7—C8—C10—C10^i^	−179.9 (3)
C1—Sn1—N2—C11	−130.1 (10)	C9—C8—C10—C10^i^	0.3 (5)
N2^i^—Sn1—N2—C11	141.7 (10)	N4^ii^—Sn2—C12—C13	−67.64 (18)
N1—Sn1—N2—C11	−36.1 (10)	N4—Sn2—C12—C13	−176.91 (18)
N1^i^—Sn1—N2—C11	−45.7 (11)	N3—Sn2—C12—C13	93.62 (17)
C12^ii^—Sn2—N3—C16	−88.13 (19)	N3^ii^—Sn2—C12—C13	21.90 (18)
C12—Sn2—N3—C16	86.48 (19)	Sn2—C12—C13—C14	173.58 (16)
N4^ii^—Sn2—N3—C16	−179.2 (2)	C12—C13—C14—C15	−177.8 (2)
N4—Sn2—N3—C16	−0.05 (18)	C20—N3—C16—C17	−0.7 (3)
N3^ii^—Sn2—N3—C16	179.6 (2)	Sn2—N3—C16—C17	179.63 (18)
C12^ii^—Sn2—N3—C20	92.20 (16)	N3—C16—C17—C18	0.2 (4)
C12—Sn2—N3—C20	−93.18 (16)	C16—C17—C18—C19	0.8 (4)
N4^ii^—Sn2—N3—C20	1.1 (3)	C17—C18—C19—C20	−1.2 (4)
N4—Sn2—N3—C20	−179.71 (16)	C17—C18—C19—C21	179.1 (2)
N3^ii^—Sn2—N3—C20	−0.09 (11)	C16—N3—C20—C19	0.3 (3)
N2^i^—Sn1—C1—C2	62.44 (19)	Sn2—N3—C20—C19	179.99 (17)
N2—Sn1—C1—C2	−42.61 (19)	C16—N3—C20—C20^ii^	−179.4 (2)
N1—Sn1—C1—C2	−134.92 (19)	Sn2—N3—C20—C20^ii^	0.3 (3)
N1^i^—Sn1—C1—C2	154.78 (19)	C18—C19—C20—N3	0.6 (3)
Sn1—C1—C2—C3	−166.65 (18)	C21—C19—C20—N3	−179.6 (2)
C1—C2—C3—C4	−176.4 (2)	C18—C19—C20—C20^ii^	−179.6 (3)
C9—N1—C5—C6	1.0 (3)	C21—C19—C20—C20^ii^	0.1 (4)
Sn1—N1—C5—C6	178.97 (18)	C20—C19—C21—C21^ii^	−0.7 (5)
N1—C5—C6—C7	0.6 (4)	C18—C19—C21—C21^ii^	179.1 (3)
C5—C6—C7—C8	−1.7 (4)		

Symmetry codes: (i) −*x*+3/2, *y*, −*z*+1/2; (ii) −*x*+3/2, *y*, −*z*+3/2.
